# Solvent and pH Stability of Poly(styrene-alt-maleic acid) (PSaMA) Membranes Prepared by Aqueous Phase Separation (APS)

**DOI:** 10.3390/membranes11110835

**Published:** 2021-10-29

**Authors:** Wouter M. Nielen, Joshua D. Willott, Wiebe M. de Vos

**Affiliations:** Membrane Surface Science (MSuS), Membrane Science and Technology Cluster, Mesa+ Institute for Nanotechnology, University of Twente, 7500 AE Enschede, The Netherlands; w.m.nielen@utwente.nl (W.M.N.); j.d.willott@utwente.nl (J.D.W.)

**Keywords:** polyelectrolytes, membranes, sustainable, responsive, aqueous phase separation

## Abstract

In the single-polyelectrolyte aqueous phase separation (APS) approach, membranes are prepared by precipitating a weak polyelectrolyte from a concentrated aqueous solution using a pH switch. This has proven to be a versatile and more sustainable method compared to conventional approaches as it significantly reduces the use of organic solvents. Poly(styrene-alt-maleic acid) (PSaMA) is a polymer that has been extensively investigated for APS and has been the basis for both open and dense membranes with good performances. These membranes are chemically crosslinked and, in this work, we further investigated ultrafiltration (UF) and nanofiltration (NF) membranes prepared with PSaMA for their stability in various organic solvents and under different pH conditions. It was shown that these membranes had stable performances in both isopropanol (IPA) and toluene, and a slightly reduced performance in N-methyl-2-pyrollidone (NMP). However, PSaMA did not perform well as a selective layer in these solvents, indicating that the real opportunity would be to use the UF-type PSaMA membranes as solvent-stable support membranes. Additionally, the membranes proved to be stable in an acidic-to-neutral pH regime (pH 2–7); and, due to the pH-responsive nature of PSaMA, for the NF membranes, a pH-dependent retention of Mg^2+^ and SO_4_^2−^ ions was observed and, for the UF membranes, a strong responsive behavior was observed, where the pH can be used to control the membrane permeability. However, long-term exposure to elevated pH conditions (pH 8–10) resulted in severe swelling of the NF membranes, resulting in defect formation, and compaction of the UF membranes. For the UF membranes, this compaction did prove to be reversible for some but not all of the membrane samples measured. These results showed that in aqueous systems, membranes prepared with PSaMA had interesting responsive behaviors but performed best at neutral and acidic pH values. Moreover, the membranes exhibited excellent stability in the organic solvents IPA and toluene

## 1. Introduction

Membrane technology is playing an increasingly large role in optimizing and improving industrial processes, making them more efficient, as well as offering new opportunities for advanced separations [1,2,3,4]. In addition, improved chemical separation membrane technology also offers solutions for environmental problems such as water scarcity [5] and increased contamination of surface waters [6,7,8]. Yet, for all the advantages membrane technology offers, it also has several disadvantages. During membrane preparation, large quantities of organic solvents are used and massive amounts of contaminated wastewater is produced [9]. Commonly used solvents are *N*-methyl pyrrolidone (NMP), dimethylformamide (DMF), and dimethylacetamide (DMAc), which are unsustainable and reprotoxic [9,10]. Therefore, it is important to not only investigate how membranes can be used to improve global sustainability or how more selective membranes can be made, but also how membranes themselves can be produced in a more sustainable way.

As of yet, a significant amount of research has already been performed toward using alternative safer and more sustainable solvents for membrane preparation [11,12,13]. This research partially focused on using less toxic solvents, for instance, dimethyl sulfoxide [14] and ionic liquids [15,16], but also on more sustainable solvents such as methyl lactate [17], and newly designed synthetic solvents [18,19]. While good membranes can be prepared using these alternative methods, they still rely on the use of organic solvents.

Aqueous phase separation (APS), a recently developed alternative method, aims to improve the sustainability of membrane preparation by avoiding the use of organic solvents [20,21]. One of the main differences with the conventional and the previously mentioned alternative approaches to prepare membranes is that APS uses polyelectrolytes instead of conventional polymers. Polyelectrolytes are highly charged polymers that are typically responsive to salt concentration and, depending on the charged group of the polyelectrolyte, also to pH [22]. Typically, polyelectrolytes are highly soluble in water due to their charged nature but, through clever use of their responsiveness membranes, can be prepared by precipitating them from aqueous solutions. There are multiple ways that this can be achieved, which are briefly discussed here. The single-polyelectrolyte APS approach uses a weak polyelectrolyte, whose charged state is pH-dependent, which is dissolved at a pH where it is charged and water-soluble and then precipitated using a pH switch to a regime where the polyelectrolyte is uncharged and insoluble in water [20,23,24,25]. Through parameters such as the pH difference between the polymer solution and the coagulation bath [20], polymer concentration [25], salt identity and concentration [24], and type of acid used [23], the precipitation can be controlled to prepare different types of membranes. Other APS approaches use a combination of two oppositely charged polyelectrolytes to prepare membranes. There are two distinct ways to this two-polyelectrolyte approach: The first method uses one weak polyelectrolyte and one strong polyelectrolyte, whose charged state is independent of pH, which are mixed at a pH in which the weak polyelectrolyte is uncharged. With a pH switch, the weak polyelectrolyte can become charged, which then induces complexation between the oppositely charged polyelectrolytes, which becomes insoluble and can be precipitated into a membrane [21,26,27,28]. Another way membranes can be prepared with the two-polyelectrolyte approach is by mixing two oppositely charged strong polyelectrolytes that are typically complex, but this complexation can be redissolved by using extreme salt concentrations. Then, by using a switch in salinity, the polyelectrolytes form again a complex and can be precipitated into a membrane [26,29,30,31,32,33]. 

This work further investigated membranes prepared with poly(styrene-alt-maleic acid) (PSaMA) using the single-polyelectrolyte APS approach. In previous works, various open and dense membranes were prepared and investigated, which displayed high retentions and good mechanical stability, demonstrating the versatility of this APS approach [23,24,25]. However, as of yet, the solvent stability of the membranes prepared with PSaMA has not been investigated. PSaMA membranes are typically chemically crosslinked with short-chain poly(ethyleneimine) PEI using a carbodiimide based crosslinking method [34] to improve the pH stability of these membranes under neutral and basic conditions where PSaMA is typically charged and water-soluble. Thus, as these membranes are crosslinked and crosslinked membranes are of interest for applications where solvent stability is required [35], it is important to investigate whether the crosslinking method for PSaMA membranes also provides solvent stability to the membranes. If PSaMA membranes are stable in various organic solvents, it would be possible to use these membranes for challenging separations under harsh conditions in industrial processes [36,37]. Additionally, as PSaMA is a weak polyelectrolyte and crosslinked with PEI, another weak polyelectrolyte, it is of interest to investigate the pH responsiveness and stability of these membranes. Herein, we demonstrated that crosslinked membranes prepared with PSaMA using the single-polyelectrolyte APS approach have good solvent stability in various organic solvents and are pH-responsive and stable under neutral and acidic conditions (pH 2–7), but problems are encountered in basic pH regimes (pH 8–10).

## 2. Experimental Procedures

### 2.1. Materials

Poly(styrene-alt-maleic acid) sodium salt solution 13% (M_w_ 350,000 g·mol^−1^, PSaMA), polyethylene glycol (M_w_ 200 g·mol^−1^, PEG 200; M_w_ 400 g·mol^−1^, PEG 400; M_w_ 600 g·mol^−1^, PEG 600; M_w_ 1500 g·mol^−1^, PEG 1500; M_w_ 2000 g·mol^−1^, PEG 2000), polyethyleneimine, branched (M_n_ 600 g·mol^−1^, PEI 600), N-(3-dimethylaminopropyl)-N′-ethylcarbodiimide hydrochloride (EDC), N-hydroxysuccinimide (NHS), magnesium sulfate, Sudan Black B, citric acid, trisodium citrate, sodium bicarbonate, glacial acetic acid, phosphoric acid 85%, and hydrochloric acid 37% were purchased from Sigma Aldrich (St. Louis, MO, USA). Ethanol 100% technical grade and isopropyl alcohol (IPA) technical grade were bought from Boom B.V. (JE Meppe, The Netherlands). *N*-Methyl-2-pyrrolidinone (NMP) 99% and *n*-hexane 99+% were purchased from Acros Organics (Waltham, MA, USA). Sodium chloride (Sanal^®^ P) was received from AkzoNobel (Amsterdam, The Netherlands). Toluene 99.8% anhydrous was purchased from ABCR (Karlsruhe, Germany). All chemicals were used as received except PSaMA, which was dried at 100 °C for up to 10 h. Coagulation baths were prepared using deionized water (DI, 1.0 µS·cm^−1^), whereas all other solutions were prepared with Milli-Q water (Millipore, Burlington, MA, USA, 0.6 μS·cm^−1^) 

### 2.2. Membrane Preparation

The membranes were prepared and crosslinked using the same methods described in our previous works and, for the readers’ convenience, the vital details are reported here [23]. PSaMA was dissolved in water with acetic acid and mixed on a roller bank. After the PSaMA was fully dissolved, Bekaert 25 µm Bekipor ST25 AL 3 steel filters were used to remove any particulate matter, and the solution was allowed to rest for at least 24 h to degas. The nanofiltration (NF) membranes were cast onto a glass substrate and prepared in 2.5 M of H_3_PO_4_, while the ultrafiltration (UF) membranes were cast on a nonwoven fabric (polyphenylene sulfide) and prepared in 0.1 M of HCl. Casting was performed using a steel casting knife with a 0.3 mm gap height and the membranes were subsequently immediately submerged into the coagulation bath. A period of 15 min after being submerged in the coagulation bath, the membranes were removed and submerged twice for 30 min in a 0.2 M HCl bath. The membranes were crosslinked with an aqueous carbodiimide-based mechanism using low-molecular-weight PEI as the crosslinker [34]. Crosslinking reactions were performed using approximately 1.04 g of EDC (5.45 mmol), 0.25 g of NHS (2.18 mmol), and 1.09 g of PEI (1.82 mmol) per 100 cm^2^ of membrane surface area at pH 5 (set with HCl). After crosslinking, membranes were washed twice for 30 min using DI water.

### 2.3. Membrane Performance Tests

The organic solvent stability tests were performed using dead-end filtration cells with a pressurized feed vessel. Membranes with 1290 mm^2^ of permeable surface area supported by nonwoven fabric (polyphenylene sulfide, resistant to all solvents used) were studied using pure water, as well as various solvents, at 1 bar of applied pressure. The pH stability measurements were performed with four crossflow cells (3650 mm^2^ of permeable surface area) with Naltex™ Alternating Strand Design feed spacers (SWM, Alpharetta, GA, USA) operated in parallel at a 0.2 m·s^−1^ crossflow velocity for measurements with NF membranes; due to limitations of the setup, 1 m·s^−1^ was used for measurements with UF membranes. During the measurement, both retentate and permeate were recycled directly into the feed except when permeate samples were taken. The pH-dependent permeability for the UF membranes was measured using 0.02 M of buffer solution using phosphate (pH 2, 7, and 8), citrate (pH 4 and 6), and carbonate (pH 9 and 10) buffers. The retention for different ions was measured with a 5 mM NaCl, 5 mM MgSO_4_ salt solution set to the desired pH using HCl and NaOH, and with an ion chromatograph (Metrohm ECO IC, Herisau, Switzerland) concentrations in the feed and permeate samples were measured. To determine the concentrations of Cl^−^ and SO_4_^2−^, a Metrosep A Supp 17—150/4.0 anion column (Metrohm, Herisau, Switzerland) with 5 mM of Na_2_CO_3_ and 0.2 mM of NaHCO_3_ as eluent at 0.9 mL·min^−1^ was used, and for Na^+^ and Mg^2+^, a Metrosep C 6—150/4.0 cation column (Metrohm, Herisau, Switzerland) with 4 mM of HNO_3_ as eluent at 0.6 mL·min^−1^ was used. The retention was determined by comparing the concentration of the permeate to that of the feed. Molecular weight cut-off measurements were performed using 1 g·L^−1^ of the various PEG molecules and analyzed with gel permeation chromatography (Agilent 1200/1260 Infinity GPC/SEC series, Polymer Standards Service data center and column compartment, Agilent, Santa Clara, CA, USA). Two Polymer Standards Service Suprema 8 mm × 300 mm columns in series: 1000 Å, 10 µm followed by 30 Å, 10 µm (PSS-Polymer Standards Service, Mainz, Germany), were used with 50 mg·L^−1^ of NaN_3_ as eluent at 1 mL·min^−1^. Concentrations were determined via refractive index detection of the feed and permeate.

### 2.4. Scanning Electron Microscopy (SEM)

SEM samples were prepared after a solvent exchange with ethanol (twice for 30 min) followed by hexane (twice for 30 min). The samples were fractured using liquid nitrogen. After at least 4 h in a vacuum oven at 30 °C, the samples were coated with 5 nm of platinum-palladium (80–20) using a Quorum Q150T ES (Quorum Technologies, Lewes, UK) and imaged with a Jeol JSM-6010LA scanning electron microscope (Jeol, Tokyo, Japan).

### 2.5. Zeta Potential 

The zeta potential measurements were performed with an Anton Paar SurPASS electrokinetic analyzer (Anton Paar, Graz, Austria). Then, 5 mM of KCl was used as an electrolyte and the streaming potential was measured at different pH values (sweeping from low to high) in an adjustable gap height cell using a 110 µM gap height. The Fairbrother and Mastin (FM) method was used for the calculation of the zeta potential [38]. 

### 2.6. pH Measurements 

The pH of the samples was measured using a Mettler Toledo FiveEasy^TM^ F20 pH meter (Mettler Toledo, Columbus, OH, USA), which was calibrated before use.

## 3. Results and Discussion

**Figure 1:**
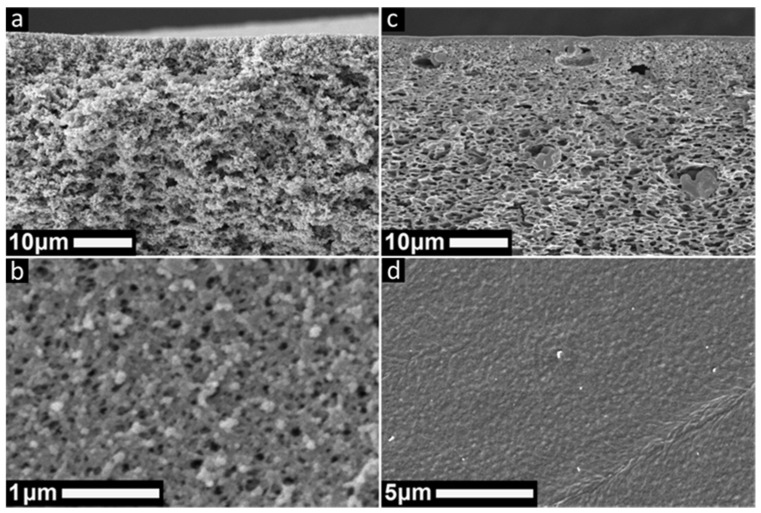
SEM cross-section and top surface images of membranes prepared in a coagulation bath with 0.1 M of HCl (**a**,**b**) or 2.5 M of H_3_PO_4_ (**c**,**d**) using a 20% *w*/*v* PSaMA, 40% *v*/*v* acetic acid polymer casting solution.

Herein, we investigated the organic solvent and pH stability of PSaMA membranes prepared with a single-polyelectrolyte APS approach. Both open ultrafiltration (UF) membranes, as well as dense nanofiltration (NF) membranes, were prepared using a casting solution containing 20% *w*/*v* PSaMA with 40% *v*/*v* acetic acid in water and crosslinked with short-chain PEI. Based on the results of our previous work, we chose to prepare open UF membranes with a mild coagulation bath of 0.1 M of HCl as this gives highly porous membranes (see Figure 1a,b). Image analysis revealed that in its dried state, the UF membrane has an average pore size 32 ± 17 nm, although a small portion of the pores are as large as 100 nm (see Figure S1 for the pore size distribution). The dense NF membrane was prepared using the same casting solution and 2.5 M of H_3_PO_4_ in the coagulation bath as this gives membranes with a thin (~300 nm), dense selective layer with few macro voids in the substructure (see Figure 1c,d) [23].

### 3.1. Organic Solvent Stability

**Figure 2:**
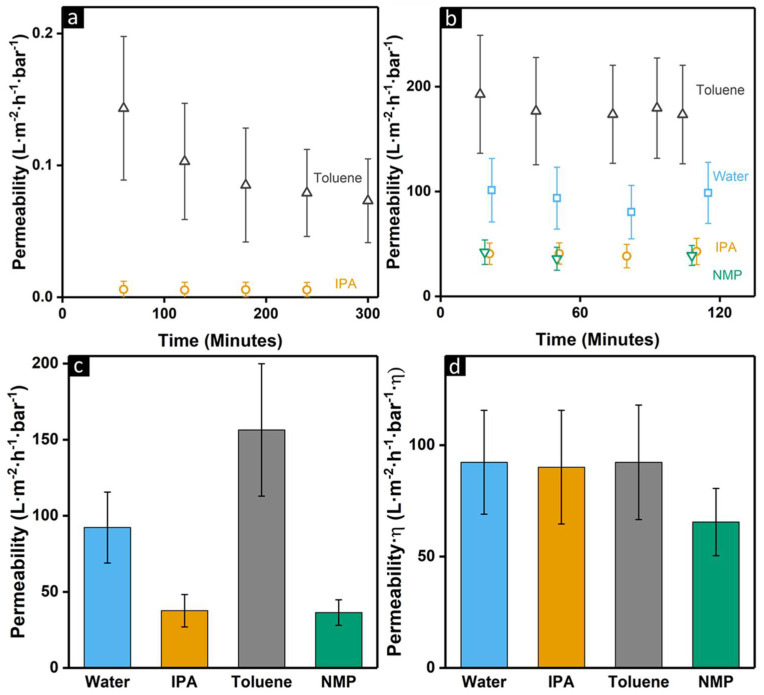
(**a**) Permeability of toluene and IPA (20 bar of applied pressure), through NF membranes prepared with a 20% *w*/*v* PSaMA with 40% *v*/*v* acetic acid polymer casting solution prepared in a 2.5 M H_3_PO_4_ coagulation bath and crosslinked with PEI. (**b**) Permeability of various solvents through UF membranes at 1 bar of applied pressure, prepared with a 20% *w*/*v* PSaMA with 40% *v*/*v* acetic acid polymer casting solution prepared in a 0.1 M HCl coagulation bath and crosslinked with PEI. (**c**) The average permeability of various solvents through UF membranes at 1 bar of applied pressure. (**d**) The average permeability of the solvents though the UF membranes at 1 bar of applied pressure multiplied by the viscosity (η) of the solvent. The error bars indicate the standard deviation of at least 3 different membrane samples.

The organic solvent stability of the membranes was investigated using the NF and UF membranes of Figure 1a,b. As crosslinking is a common method to improve the solvent stability of membranes [35], it is thus expected that these membranes are, to a certain extent, resistant to exposure to organic solvents. To test the solvent resistance, toluene, IPA, and NMP were chosen, where toluene has the lowest polarity index and NMP the highest [39]. In Figure 2a, the permeability of toluene and IPA through the NF membranes is shown, which is observed to be stable after initial compaction, but very low. As the toluene permeability is significantly higher than that of IPA, a retention experiment in toluene was performed using a small dye molecule (Sudan Black B, 456.5 Dalton). It was found that there is no retention of the dye, which is surprising as measurements in water showed that these membranes have a molecular weight cut-off of 220 ± 20 Dalton (Figure S2a). Therefore, it is highly likely that the measured high permeability for toluene is predominantly caused by defects in the selective layer. As the membrane shrinks ~5% during the solvent exchange to toluene, it is expected that, due to the stress of shrinking, defects in the selective layer are formed; these defects also explain the large error bars observed as the number of defects likely differ per sample. Regardless of the low permeability and defects, the membranes have a relatively stable performance in both IPA and toluene at 20 bar of applied pressure, which is more than enough for most NF applications [1]. This shows that PSaMA is simply not a material that is useful as the selective layer for the filtration of these solvents, but that it does have good mechanical stability in these solvents.

To further investigate the solvent stability of crosslinked PSaMA membranes, the UF membranes were tested. As seen in Figure 2bc, significant differences in permeability are observed depending on the solvent. However, as permeability is inversely related to viscosity (η), it is important to take the difference in viscosity of the solvents into account [40]. When normalized for viscosity differences (Figure 2d), the IPA and toluene permeability are the same as the water permeability, demonstrating that these membranes are completely stable in those solvents over a measured period of 2 h. For NMP, this is not completely the case as, even when the viscosity is taken into account, the permeability is somewhat lower than those of the other solvents. However, while the permeability is lower, it does remain stable during the measurement, indicating a small degree of compaction instead of instability of the membrane. To test whether this is an effect of swelling or shrinking of the membrane, the water permeability was measured again after the NMP filtration, which resulted in a stable but similarly reduced permeability. This indicates that the membranes were irreversibly compacted during the NMP filtration experiment. It is expected that as NMP is typically a very good solvent for polymers and due its high polarity, it can enhance the mobility of the PSaMA polymer chains where it is not highly crosslinked, which results in compaction of the membrane. When the SEM images from after the solvent filtration were compared to those from before, no significant changes were observed (Figure S3a–d). This indicates that the membranes are mostly stable in NMP, and it is expected that it is possible to achieve full stability in NMP by, for instance, increasing the crosslink density. 

The stability of these membranes in the various organic solvents demonstrates that it is possible to use these membranes for demanding applications involving harsh organic solvents. These membranes can be used as they are but can also be an interesting substrate for interfacial polymerization or (polyelectrolyte) dip coating to prepare high-performance thin-film composite membranes for organic solvent nanofiltration applications. 

### 3.2. pH Responsiveness and Stability

**Figure 3:**
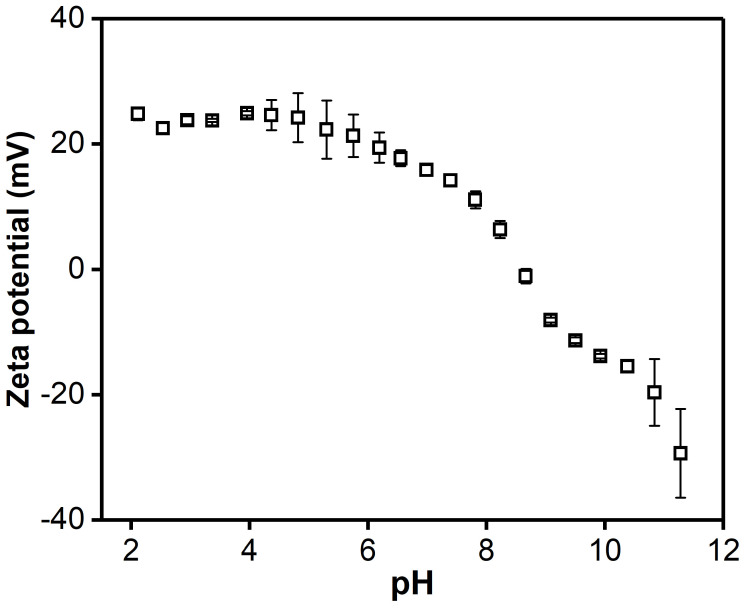
Zeta potential of the PSaMA NF membranes at different pH values calculated using the Fairbrother and Mastin (FM) method measured from low to high pH values. The error bars indicate the standard deviation of 4 measurements on a single membrane sample.

As PSaMA is a pH-responsive polymer and the membranes are crosslinked with short-chain poly(ethyleneimine) (PEI), another pH-responsive polymer, it is natural to assume that the resultant membrane has some sort of a pH-responsive behavior. To measure the extent of this pH-responsiveness, zeta potential measurements were performed using the PSaMA NF membranes. As seen in Figure 3, at a low pH, the surface is positively charged, which is logical as the carboxylic acid groups are protonated and uncharged, while the amine groups of the PEI are also protonated and, therefore, positively charged. As the pH is increased, the measured zeta potential decreases until it becomes negative at pH 8.5. This matches with the pK_a_ values of PSaMA, which are approximately 4.5 and 9 [41] but are somewhat surprising as one would expect that at pH 6, already a large amount of the PSaMA is negatively charged and, thus, the membrane as well. That the zeta potential only becomes negative around pH 8.5, which is close to the pK_a_ values of PEI (8.18–9.94) [42], indicates that PEI has a strong effect on the surface charge. This is not entirely surprising, as, during the crosslinking of the PSaMA membranes with PEI, there is also a large possibility that PEI is grafted onto the top layer of the membrane and, therefore, has a large influence on the surface properties of the membrane. At a pH above 8.5, the zeta potential becomes increasingly more negative, indicating more groups are deprotonated, meaning PEI becomes uncharged and the negative charges of PSaMA start to dominate the membranes’ net charge. At a pH above 10, a problem is encountered as the membranes start to swell significantly, which results in unreliable data. This swelling irreversibly damages the membrane and is most likely caused by the large number of negative charges of the carboxylate groups of PSaMA. It is expected that, due to the very high local concentration of negative charges (approximately 5 M if PSaMA is fully charged), the electrostatic repulsion and osmotic pressure become so strong that it either partially breaks the crosslinking or forces a polymer rearrangement, which results in a loss of structural integrity of the membrane.

**Figure 4:**
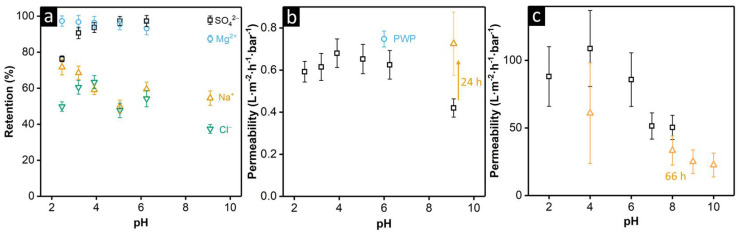
(**a**) Retention of various ions at different feed solution (5 mM of MgSO_4_ plus 5 mM of NaCl) pH values by the PSaMA NF membranes. (**b**) Permeability of the PSaMA NF membranes under the different pH conditions from (**a**), and the pure water permeability (PWP) measured with deionized water. At pH 9.1, the permeability had significantly changed after 24 h, shown by the second data point at pH 9.1 (Δ). (**c**) The water permeability of the PSaMA UF membranes of 0.02 M buffer solutions at different pH values measured over several hours from low to high pH, after which the permeability at pH 4 was measured again. At pH 8, a long-term experiment (66 h) was performed, after which reduced permeability values were observed (Δ). The error bars indicate the standard deviation of measurements from 4 different membrane samples.

To further investigate the pH stability and responsiveness of the PSaMA NF membranes, the permeability and ion retention were measured at different pH values, ranging from pH 2.5 to 10 (see Figure 4a,b). In the low pH range (2.5–3.3), a high retention for the Mg^2+^ is observed and a low retention for SO_4_^2−^, which is expected as the zeta potential measurements showed that the membrane has a positive charge in that range and is, thus, based on Donnan exclusion species with the same charge as the membranes are retained more than oppositely charged ones are [43]. However, as the retention of SO_4_^2−^ is still relatively high, it is highly likely that, besides Donnan exclusion, dielectric and size exclusion plays a large part in the ion retention [44]. It is very interesting that, especially at a feed pH of 2.5, the retention for both Mg^2+^ and Na^+^ is much higher than that of the anions. This means that, as charge neutrality must be maintained, a significant amount of H^+^ has to permeate through the membrane, indicating that these membranes are selective for H^+^ over Na^+^. It is not unexpected that H^+^ can permeate more easily through the membrane than Na^+^ can, as the Na^+^ ions have to physically permeate through the entire membrane, while the H^+^ ions can easily hop between water molecules and functional groups [45]. The increased H^+^ permeation was confirmed by pH measurements, which showed that, while the feed pH was stable at 2.5, the pH of the permeate samples reduced to 2.4 (~26% increase in H^+^ concentration). At higher feed pH values, the retention of Mg^2+^ decreased, while the retention of SO_4_^2−^ increased, which fits with the pH-responsive behavior of PSaMA, which became more negatively charged. What is interesting is that in the zeta potential measurements, the largest differences were observed when the pH was higher than 6, while in the ion retention experiment, the largest differences were seen below pH 6. An explanation is that the zeta potential only measures the surface charge of the membrane, which is strongly influenced by the presence of grafted PEI chains. For the ion retention, the charge inside the selective layer is more important, which is determined by PSaMA, the membrane material itself, as, due to the low-molecular-weight cut-off of the membrane, it is unlikely that there is a large amount of PEI (M_n_ 600) inside the selective layer. It is expected that PEI is predominantly present in the porous support structure and the top surface of the membrane. Therefore, the charge inside the selective layer is much more dependent on PSaMA instead of PEI. 

With a feed pH value higher than 6.5, difficulties were encountered as unbuffered salt solutions were used for the retention measurements as the presence of buffer would affect the salt retention; therefore, the buffer capacity of the membranes themselves and CO_2_ absorption from the atmosphere reduced the pH values during the measurement. During the measurements with the NF membranes, a feed with the desired pH value was permeated with the permeate recycled into the feed for 16 h to equilibrate the membranes, after which a fresh feed with the desired pH was used for the measurement. For pH values of pH 6.5 and lower, this provided a stable pH and performance throughout the measurement; however, at higher pH values, significant discrepancies were observed. When a feed pH of 8 was used, the pH quickly dropped to 6.5 due to the membranes’ buffer capacity and dissolved CO_2_. When a feed solution at pH 10 was used, it was observed that while the pH of the feed solution was slowly decreasing, the ion retention followed the same trend as before where the SO_4_^2−^ retention was slightly increased and the Mg^2+^ slightly decreased measured when the feed pH was at 9.1. Interestingly, the pH of the permeate samples was still at 6.5 during this measurement, indicating the membrane itself was buffering the pH of the permeate. After another 24 h of permeating this feed solution, a significant change was observed as the permeability was strongly increased and the ion retentions severely decreased. When molecular weight cut-off measurements from after the high pH exposure were compared to those before, strong differences were observed (see Figure S2). That after high pH exposure, a maximum of only 75% retention was achieved regardless of the molecular weight, indicates the presence of newly formed defects (Figure S2b).

Further investigation into the pH stability of PSaMA membranes was performed by measuring the permeability of the UF membranes in 0.02 M buffer solutions at different pH values (see Figure 4c). After initial compaction, steady permeability values were measured over several hours at each of the different pH values measured from low to high pH. At pH 8, a 66 h experiment was performed, which revealed that the permeability significantly decreased over time, which can either be due to instability of the membrane at these pH values, leading to additional compaction, or due to fouling. After the permeabilities at pH 9 and 10 were measured, the permeability was again measured at pH 4, which was significantly lower than before, indicating an irreversible reduction of flux. It is, however, important to note that, as seen by the increased error bars, the variation between the different membrane sample was large and that one of the four measured samples recovered approximately 96% of its original flux at pH 4. This means that, while most membranes were irreversibly compacted during the long-term exposure to pH 8 and higher, it was possible for these membranes to be stable under these pH conditions. A possible explanation for the variation in stability between the different membranes is that even though the same conditions were used, the crosslinking density was different, and with insufficient crosslinking, the membrane structure was less stable. However, when SEM images were taken of the different membranes after the permeation experiments, apart from a small amount of biofouling, no significant structural differences were observed (see Figure S3e,f).

Comparing the NF and UF membranes performances, it was observed that both have an optimum in permeability at pH 4 and that, at pH 7 and higher, the permeability is significantly lower. This demonstrates that membranes prepared from the responsive PSaMA indeed retain their relevant responsive behavior, allowing control over permeability and separation properties. At pH 8–10, it was observed that, while the NF membranes show severe performance issues and defects are formed, the UF membranes only have a reduction in flux, which, for some of the membranes, is reversible. It is expected that this is caused by the swelling of PSaMA, which becomes more charged under these pH conditions, which, for the NF membranes, results in defect formation in the dense selective layer, but as with the UF membranes, as there is no dense layer, the effect is less severe and only leads to some compaction of the membrane. 

## 4. Conclusions

UF and NF membranes were prepared with PSaMA using the single-polyelectrolyte APS approach. While the performances of these membranes in aqueous systems at a neutral pH have been extensively investigated in previous studies [23,24,25], their stability in organic solvents (IPA, toluene, NMP) and different pH values (acidic and alkaline) has not been reported. In IPA and toluene, the membranes showed excellent stability even at high pressures, while in NMP, a slight reduction in the viscosity-normalized permeability was observed compared to the other solvents. This showed that PSaMA did not perform well as a selective layer in these solvents, indicating that the real opportunity would be to use the UF-type PSaMA membranes as solvent-stable support membranes. Investigations into the pH stability and responsiveness of PSaMA membranes showed that the NF membranes had a fully stable performance between pH 2.5 and 6.5 and that the retention of divalent ions was pH-dependent. At pH 2.5, the higher Mg^2+^ retention was measured (97% ± 3%), while the SO_4_^2−^ retention (76% ± 2%) was at its lowest. At pH 6.5, a lower Mg^2+^ retention (93% ± 4%) and a significantly higher SO_4_^2−^ retention (97% ± 3%) were measured. At higher pH values, severe swelling issues were encountered, which resulted in defect formation in the dense selective layer and, therefore, a loss of performance for the NF membranes. The UF membranes showed very relevant responsive behavior, where the pH can be used to control the membrane permeability, with the highest permeability at pH 4 (109 ± 28 L·m^−2^·h^−1^·bar^−1^) and lowest at pH 10 (23 ± 9 L·m^−2^·h^−1^·bar^−1^). Additionally, it was observed that long-term exposure to pH conditions of 8 or higher led to compaction of the membranes, which was reversible for one of the measured membrane samples but irreversible for others. This demonstrated that crosslinked membranes prepared with PSaMA using the single-polyelectrolyte APS approach were stable in various organic solvents and showed relevant pH-responsive behavior under aqueous conditions. The membranes did lose some of their stability at higher pH conditions and, in NMP, this indicates it is important to further investigate the crosslinking procedure to increase the crosslinking density and optimize the stability of these membranes.

## Data Availability

Not applicable.

## Supplementary Materials

The following are available online at https://www.mdpi.com/article/10.3390/membranes11110835/s1, Figure S1. Pore size distribution of the UF membranes prepared in a coagulation bath with 0.1 M of HCl using a 20% *w*/*v* PSaMA, 40% *v*/*v* acetic acid polymer casting solution. Pore sizes were analyzed using ImageJ software. Figure S2. Molecular weight cut-off of the NF membranes prepared in a coagulation bath with 2.5 M of H3PO4 using a 20% *w*/*v* PSaMA, 40% *v*/*v* acetic acid polymer casting solution before exposure to a pH 10 feed solution for 48 h (a) and after (b). Figure S3. SEM cross-section and top surface images of membranes prepared in a coagulation bath with 0.1 M of HCl using a 20% *w*/*v* PSaMA, 40% *v*/*v* acetic acid polymer casting solution before (a,b), after (c,d) solvent filtration with IPA, toluene, and NMP, and after (e,f) filtration under different pH conditions for 7 days.
