# Supplementary material for: Solvent and pH Stability of Poly(styrene-alt-maleic acid) (PSaMA) Membranes Prepared by Aqueous Phase Separation (APS)
Source: Membranes (Basel). 2021 Oct 29;11(11):835. doi: 10.3390/membranes11110835 (PMC8624750; doi:10.3390/membranes11110835)
Supplement: Supplementary file 1 [file membranes-11-00835-s001.zip › membranes-1431031-supplementary.pdf]

Pore size distribution

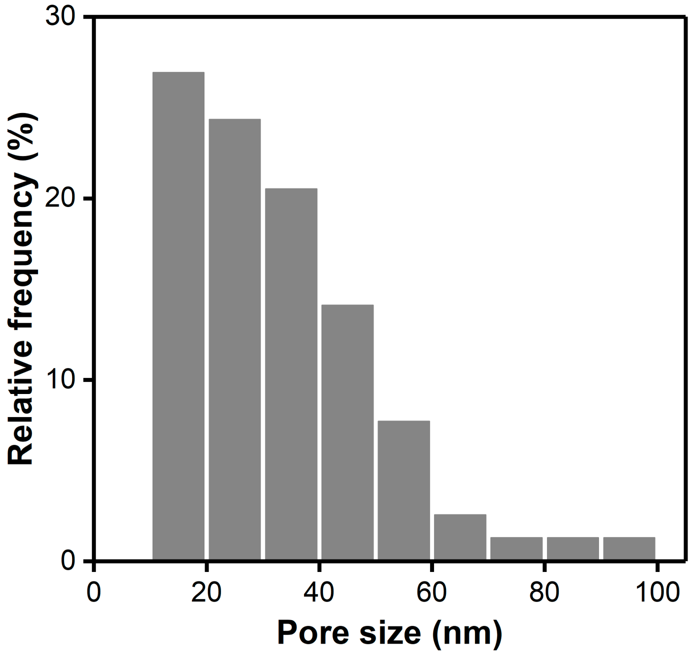

Figure S1. Pore size distribution of the UF membranes prepared in a coagulation bath with 0.1 M HCl using a 20% w/v PSaMA, 40% v/v acetic acid polymer casting solution. Pore sizes were analyzes using ImageJ software.

Molecular weight cut-off

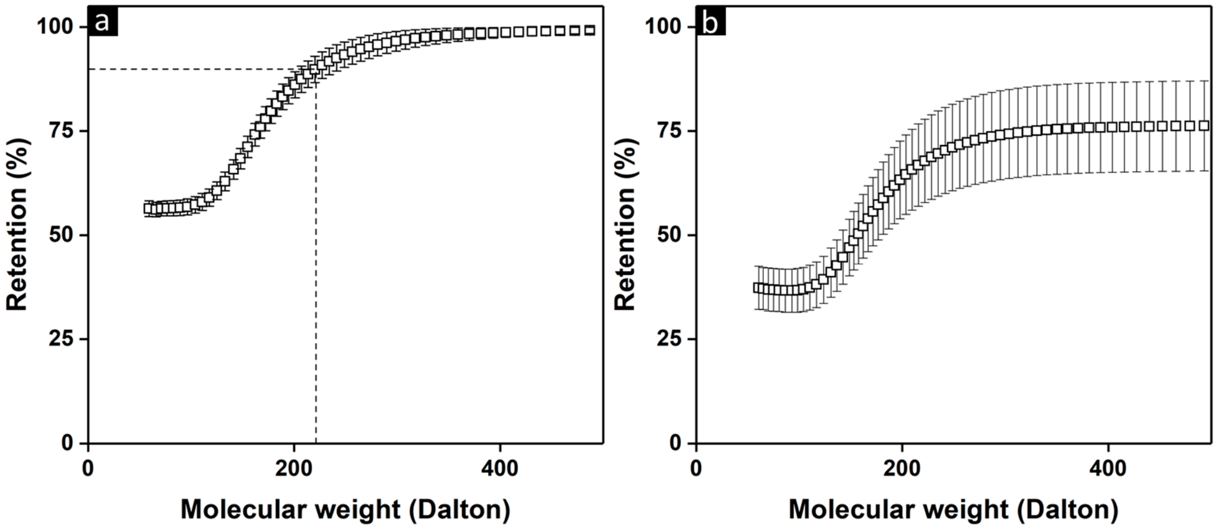

Figure S2. Molecular weight cut-off of the NF membranes prepared in a coagulation bath with 2.5 M H_3PO_4 using a 20% w/v PSaMA, 40% v/v acetic acid polymer casting solution before exposure to a pH 10 feed solution for 48 hours (a) and after (b).

SEM images

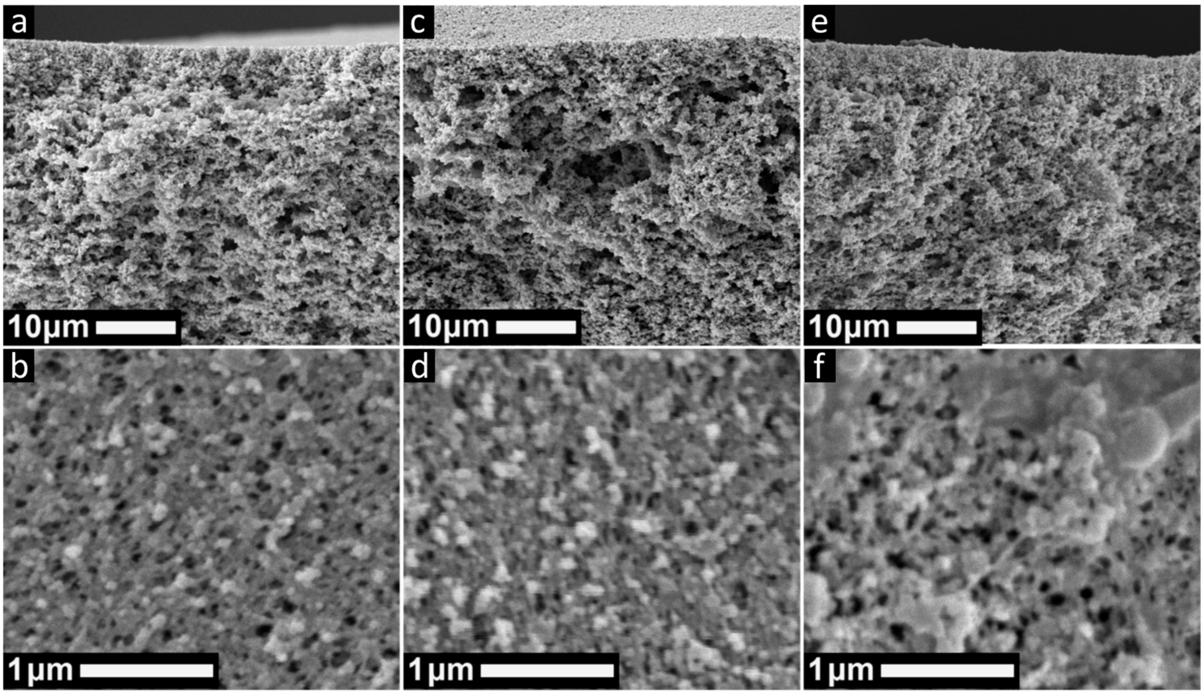

Figure S3. SEM cross section and top surface images of membranes prepared in a coagulation bath with 0.1 M HCl using a 20% w/v PSaMA, 40% v/v acetic acid polymer casting solution before (a,b), after (c,d) solvent filtration with IPA, toluene, and NMP, and after (e, f) filtration at different pH conditions for 7 days.
